# Supplementary material for: Incidence and predictors of acute kidney injury among adults admitted to the medical intensive care unit of a Comprehensive Specialized Hospital in Central Ethiopia
Source: PLoS One. 2024 Jun 26;19(6):e0304006. doi: 10.1371/journal.pone.0304006 (PMC11207181; doi:10.1371/journal.pone.0304006)
Supplement: S1 Table — (DOCX) [file pone.0304006.s001.docx]

The supplementary file 1: shows that stratified analysis after forming two strata (with invasive ventilation and without invasive ventilation) by keeping the others variables in the multivariate analysis.

| Variables | Haz. Ratio | P- Value | 95% CI | |
| --- | --- | --- | --- | --- |
|  |  |  | lower | Upper |
| Comorbidity |  |  |  |  |
| yes | 1.009157 | 0.965 | .6686174 | 1.523139 |
| Hypertension |  |  |  |  |
| yes | 1.768001 | 0.007 | 1.172301 | 2.666403 |
| Vancomycine |  |  |  |  |
| yes | 1.914866 | 0.016 | 1.128493 | 3.249212 |
| Hospital acquired infection |  |  |  |  |
| yes | 1.590513 | 0.052 | .9950606 | 2.542289 |
| Peak Inspiratory Pressure |  |  |  |  |
| >=35cmH2o | 1.313513 | 0.209 | .8582472 | 2.010279 |
| Positive end-expiratory pressure |  |  |  |  |
| >10cmH2o | 1.415183 | 0.134 | .8991072 | 2.22748 |
| Respiratory Rate |  |  |  |  |
| >=24b/m | 1.059066 | 0.818 | .6498496 | 1.725971 |
| Fluid Balance |  |  |  |  |
| Negative | **1.602848** | 0.030 | 1.045643 | 2.456979 |
| Vasopressor |  |  |  |  |
| yes | 2.269471 | 0.000 | 1.48676 | 3.464242 |
| Mean Arterial Blood Pressure |  |  |  |  |
| >=65mmHg | 1.116599 | 0.556 | .7735699 | 1.61174 |
