# Supplementary material for: Incidence and predictors of acute kidney injury among adults admitted to the medical intensive care unit of a Comprehensive Specialized Hospital in Central Ethiopia
Source: PLoS One. 2024 Jun 26;19(6):e0304006. doi: 10.1371/journal.pone.0304006 (PMC11207181; doi:10.1371/journal.pone.0304006)
Supplement: Attachment — Submitted filename: PONE-D-24-02557_reviewer.pdf [file pone.0304006.s004.pdf]

PLOS ONE

Incidence and Predictors of Acute Kidney Injury among Patients in Intensive Care Unit at a Comprehensive Specialized Hospital in Central Ethiopia

--Manuscript Draft--

**Manuscript Number:**	PONE-D-24-02557
**Article Type:**	Research Article
**Full Title:**	Incidence and Predictors of Acute Kidney Injury among Patients in Intensive Care Unit at a Comprehensive Specialized Hospital in Central Ethiopia
**Short Title:**	Incidence and Predictors of Acute Kidney Injury among Patients in Intensive Care Unit
**Corresponding Author:**	Taye Mezgebu Ashine, MSc<br>WCU: Wachemo University<br>Hosanna, ETHIOPIA
**Keywords:**	Acute kidney injury<br>incidence<br>Intensive care unit<br>predictors<br>Ethiopia
**Abstract:**	ABSTRACT<br>Background: Acute kidney injury (AKI) is a common complication of the Intensive Care Unit. Globally, AKI is a major public health concern. It affects 13 million people and, as a consequence, almost two million deaths worldwide. AKI among critically ill ICU patients is associated with increased morbidity and mortality. The study aimed to assess Acute Kidney Injury incidence and associated factors among patients admitted to the Intensive Care Unit.<br>Method: A retrospective cohort study was conducted by reviewing charts of 317 systematically selected patients admitted to the ICU from September 1, 2018 to August 30, 2022 in Wachemo University Nigist Ellen Mohammed Memorial Comprehensive Specialized Hospital. The extraction tool was used for the data collection, Epi-data version 4.6.0 for data entry, and STATA version 14 for data cleaning and analysis. The Kaplan-Meier, log-rank test, and life table were used to describe the data. The Cox proportional hazard regression model was used for analysis.<br>Results: The incidence rate of AKI was 30.1 (95% CI: 25.33, 35.8) per 1000 person-days observation, with a median survival time of 23 days. It was found that patients with invasive mechanical ventilation (AHR = 2.64; 95% CI: 1.46-4.78), negative fluid balance (AHR = 2.00; 95% CI: 1.30-3.03), hypertension (AHR = 1.6; 95% CI: 1.05-2.38), and a vasopressor (AHR = 1.72; 95% CI: 1.10-2.63) were independent predictors of acute kidney injury.<br>Conclusion: The incidence of AKI was a major concern in the ICU of the study area. In the intensive care unit (ICU), it was found that patients with vasopressors, invasive mechanical ventilation, negative fluid balance, and chronic hypertension were independent predictors of developing AKI. It would be better if clinicians in the ICU provided targeted interventions through close monitoring and evaluation of those patients with invasive ventilation, chronic hypertension, negative fluid balance, and vasopressors.
**Order of Authors:**	Taye Mezgebu Ashine, MSc
	Migbar Sibhat Mekonnen, MSc
	Asnakech Zekiwos Heliso, MSc
	Yesuneh Dejene Wolde, MSc
	Getachew Ossabo Babore, MSc
	Zerihun Demisse Bushen, MSc
	Elias Ezo Ereta, MSc
	Sentayehu Admasu Saliya, MSc

Powered by Editorial Manager® and ProduXion Manager® from Aries Systems Corporation

	Bethelhem Birehanu Muluneh, MSc
	Samrawit Ali Jemal, MSc
**Additional Information:**	
**Question**	**Response**
**Financial Disclosure**<br><br>Enter a financial disclosure statement that describes the sources of funding for the work included in this submission. Review the submission guidelines for detailed requirements. View published research articles from *PLOS ONE* for specific examples.<br><br>This statement is required for submission and **will appear in the published article** if the submission is accepted. Please make sure it is accurate.<br><br>**Funded studies**<br>Enter a statement with the following details:<br>• Initials of the authors who received each award<br>• Grant numbers awarded to each author<br>• The full name of each funder<br>• URL of each funder website<br>• Did the sponsors or funders play any role in the study design, data collection and analysis, decision to publish, or preparation of the manuscript?<br><br>Did you receive funding for this work?	Yes
Please add funding details.<br>as follow-up to "**Financial Disclosure**<br><br>Enter a financial disclosure statement that describes the sources of funding for the work included in this submission. Review the submission guidelines for detailed requirements. View published research articles from *PLOS ONE* for specific examples.<br><br>This statement is required for submission and **will appear in the published article** if the submission is accepted. Please make	This original research was funded by Wachemio University for data collection and analysis in the 2023 academic year of the university.

Powered by Editorial Manager® and ProduXion Manager® from Aries Systems Corporation

sure it is accurate.<br><br>**Funded studies**<br>Enter a statement with the following details:<br>• Initials of the authors who received each award<br>• Grant numbers awarded to each author<br>• The full name of each funder<br>• URL of each funder website<br>• Did the sponsors or funders play any role in the study design, data collection and analysis, decision to publish, or preparation of the manuscript?<br><br>Did you receive funding for this work?"	
Please select the country of your main research funder (please select carefully as in some cases this is used in fee calculation).<br>as follow-up to "**Financial Disclosure**<br><br>Enter a financial disclosure statement that describes the sources of funding for the work included in this submission. Review the submission guidelines for detailed requirements. View published research articles from *PLOS ONE* for specific examples.<br><br>This statement is required for submission and **will appear in the published article** if the submission is accepted. Please make sure it is accurate.<br><br>**Funded studies**<br>Enter a statement with the following details:<br>• Initials of the authors who received each award<br>• Grant numbers awarded to each author<br>• The full name of each funder<br>• URL of each funder website<br>• Did the sponsors or funders play any role in the study design, data collection and analysis, decision to publish, or preparation of the manuscript?<br><br>Did you receive funding for this work?"	ETHIOPIA - ET

**Competing Interests**<br><br>Use the instructions below to enter a competing interest statement for this submission. On behalf of all authors, disclose any competing interests that could be perceived to bias this work—acknowledging all financial support and any other relevant financial or non-financial competing interests.<br><br>This statement is required for submission and **will appear in the published article** if the submission is accepted. Please make sure it is accurate and that any funding sources listed in your Funding Information later in the submission form are also declared in your Financial Disclosure statement.<br><br>View published research articles from *PLOS ONE* for specific examples.<br><br>**NO authors have competing interests**<br><br>Enter: *The authors have declared that no competing interests exist.*<br><br>**Authors with competing interests**<br><br>Enter competing interest details beginning with this statement:<br><br>*I have read the journal's policy and the authors of this manuscript have the following competing interests: [insert competing interests here]*<br><br>* typeset	The authors declared no conflict of interest
**Ethics Statement**<br><br>Enter an ethics statement for this submission. This statement is required if the study involved:<br><br>• Human participants<br>• Human specimens or tissue<br>• Vertebrate animals or cephalopods	This study was approved by the Institution of Review Board (IRB) of Wachemo University. The study was performed in accordance with the Declaration of Helsinki. The documentation of informed consent was waived by our institutional review board at Wachemo University.

Powered by Editorial Manager® and ProduXion Manager® from Aries Systems Corporation

• Vertebrate embryos or tissues<br>• Field research<br><br>Write "N/A" if the submission does not require an ethics statement.<br><br>General guidance is provided below. Consult the submission guidelines for detailed instructions. **Make sure that all information entered here is included in the Methods section of the manuscript.**	

Powered by Editorial Manager® and ProduXion Manager® from Aries Systems Corporation

**Format for specific study types**<br><br>**Human Subject Research (involving human participants and/or tissue)**<br>• Give the name of the institutional review board or ethics committee that approved the study<br>• Include the approval number and/or a statement indicating approval of this research<br>• Indicate the form of consent obtained (written/oral) or the reason that consent was not obtained (e.g. the data were analyzed anonymously)<br><br>**Animal Research (involving vertebrate animals, embryos or tissues)**<br>• Provide the name of the Institutional Animal Care and Use Committee (IACUC) or other relevant ethics board that reviewed the study protocol, and indicate whether they approved this research or granted a formal waiver of ethical approval<br>• Include an approval number if one was obtained<br>• If the study involved *non-human primates*, add *additional details* about animal welfare and steps taken to ameliorate suffering<br>• If anesthesia, euthanasia, or any kind of animal sacrifice is part of the study, include briefly which substances and/or methods were applied<br><br>**Field Research**<br><br>Include the following details if this study involves the collection of plant, animal, or other materials from a natural setting:<br>• Field permit number<br>• Name of the institution or relevant body that granted permission	
**Data Availability**<br><br>Authors are required to make all data underlying the findings described fully available, without restriction, and from the time of publication. PLOS allows rare exceptions to address legal and ethical concerns. See the PLOS Data Policy and FAQ for detailed information.	Yes - all data are fully available without restriction

A Data Availability Statement describing where the data can be found is required at submission. Your answers to this question constitute the Data Availability Statement and **will be published in the article**, if accepted.<br><br>**Important:** Stating 'data available on request from the author' is not sufficient. If your data are only available upon request, select 'No' for the first question and explain your exceptional situation in the text box.<br><br>Do the authors confirm that all data underlying the findings described in their manuscript are fully available without restriction?	
**Describe where the data may be found in full sentences. If you are copying our sample text, replace any instances of XXX with the appropriate details.**<br><br>• If the data are **held or will be held in a public repository**, include URLs, accession numbers or DOIs. If this information will only be available after acceptance, indicate this by ticking the box below. For example: *All XXX files are available from the XXX database (accession number(s) XXX, XXX.*).<br>• If the data are all contained **within the manuscript and/or Supporting Information files**, enter the following: *All relevant data are within the manuscript and its Supporting Information files.*<br>• If neither of these applies but you are able to provide **details of access elsewhere**, with or without limitations, please do so. For example:<br><br>*Data cannot be shared publicly because of [XXX]. Data are available from the XXX Institutional Data Access / Ethics Committee (contact via XXX) for researchers who meet the criteria for access to confidential data.*<br><br>*The data underlying the results presented in the study are available from (include the name of the third party*	Extra data that support the findings of this study are accessible from the corresponding author upon reasonable request and can be shared upon legal request via tayemezgebu26@gmail.com

and contact information or URL).<br>• This text is appropriate if the data are owned by a third party and authors do not have permission to share the data.<br><br>* typeset	
Additional data availability information:	Tick here if the URLs/accession numbers/DOIs will be available only after acceptance of the manuscript for publication so that we can ensure their inclusion before publication.

Powered by Editorial Manager® and ProduXion Manager® from Aries Systems Corporation

Manuscript Click here to access/download;Manuscript;Manuscript.docx

Incidence and Predictors of Acute Kidney Injury among Patients in Intensive Care Unit at a Comprehensive Specialized Hospital in Central Ethiopia

Taye Mezgebu Ashine[1*], Migbar Sibhat Mekonnen[2], Asnakech Zekiwos Heliso[1], Yesuneh Dejene Wolde[3], Getachew Ossabo Babore[1], Zerihun Demisse Bushen[4], Elias Ezo Ereta[1], Sentayehu Admasu Saliya[1] Bethelhem Birehanu Muluneh[4], and Samrawit Ali J emal[3]

[1]Department of Nursing, College of Medicine and Health Science, Wachemo University, Hosanna, Ethiopia

[2]Department of Pediatric and Child Health Nursing, College of Medicine and Health Science, Dilla University, Dilla, Ethiopia

[3] Department of Midwifery, College of Medicine and Health Science, Wachemo University, Hosanna, Ethiopia

[4] Department of Pediatric and Child Health Nursing, College of Medicine and Health Science, Wachemo University, Hosanna, Ethiopia

Corresponding Author: Taye Mezgebu Ashine:- tayemezgebu26@gmail

ABSTRACT

Background: Acute kidney injury (AKI) is a common complication of the Intensive Care Unit. Globally, AKI is a major public health concern. It affects 13 million people and, as a consequence, almost two million deaths worldwide. AKI among critically ill ICU patients is associated with increased morbidity and mortality. The study aimed to assess Acute Kidney Injury incidence and associated factors among patients admitted to the Intensive Care Unit.

Method: A retrospective cohort study was conducted by reviewing charts of 317 systematically selected patients admitted to the ICU from September 1, 2018 to August 30, 2022 in Wachemo University Nigist Ellen Mohammed Memorial Comprehensive Specialized Hospital. The extraction tool was used for the data collection, Epi-data version 4.6.0 for data entry, and STATA version 14 for data cleaning and analysis. The Kaplan-Meier, log-rank test, and life table were used to describe the data. The Cox proportional hazard regression model was used for analysis.

Results: The incidence rate of AKI was 30.1 (95% CI: 25.33, 35.8) per 1000 person-days observation, with a median survival time of 23 days. It was found that patients with invasive mechanical ventilation (AHR = 2.64; 95% CI: 1.46-4.78), negative fluid balance (AHR = 2.00; 95% CI: 1.30-3.03), hypertension (AHR = 1.6; 95% CI: 1.05-2.38), and a vasopressor (AHR = 1.72; 95% CI: 1.10-2.63) were independent predictors of acute kidney injury.

Conclusion: The incidence of AKI was a major concern in the ICU of the study area. In the intensive care unit (ICU), it was found that patients with vasopressors, invasive mechanical ventilation, negative fluid balance, and chronic hypertension were independent predictors of developing AKI. It would be better if clinicians in the ICU provided targeted interventions through close monitoring and evaluation of those patients with invasive ventilation, chronic hypertension, negative fluid balance, and vasopressors.

Keywords: Acute kidney injury, incidence, Intensive care unit, predictors, Ethiopia

Introduction

Acute kidney injury (AKI) is characterized by an abrupt decrease in kidney function that occurs within hours and encompasses both structural damage and loss of function[1]. The glomerular filtration rate is commonly used as the best overall index of kidney function in critically ill patients[2, 3]. Acute kidney injury is a common problem in both adult and pediatric populations associated with increased morbidity and mortality[4, 5]. AKI is a common complication in the Intensive Care Unit [6, 7]. It is one of the most common risk factors for increased mortality in the ICU[8].

Globally, it is a major public health concern and can be variable between high- and low-income nations. It affects more than 13 million population, and results in 1.7 million deaths each year worldwide[9, 10]. It is a serious life-threatening disease and a global burden in both underdeveloped and developed countries. A study conducted in the USA found that 1.2 million people per year develop AKI during the hospital stay, of which 300,000 people die annually[11]. Moreover, studies showed that the prevalence of AKI IN the ICU is extremely variable between 1% to 66%[4]. Various published studies revealed that high incidence of AKI in the intensive care unit, in South Africa at 58.5%[12], Tanzania at 55.3%[13], Sudan at 39.0%[14], Egypt at 37.4%[15], China 51.0%[16], Norway 38%[17], Brazil 40.3%[18], and Jordan 31.6%[19]. Once developed, AKI is associated with increased death rates amongst critically sick patients, prolongs hospital stays, and is highly expensive to manage clinical costs[20].

Several reviewed kinds of literature demonstrated multifactorial risk factors associated with AKI, and the aetiology and risk factors for acute kidney injury (AKI) vary geographically. These include acute tubular necrosis, Socio-demographic Characteristics, environmental factors, dehydration, trauma, and sepsis from fulminant infections, which can include malaria, tuberculosis, gastroenteritis, diabetic mellitus, hypertension, obesity, cardiovascular diseases, hypovolemia, and opportunistic infections common in HIV/AIDS patients, among other conditions[21-26].

AKI is a common issue everywhere that arises in a variety of contexts and hurts patient outcomes, particularly for ICU patients. In developing countries like Ethiopia, data on the incidence, risk factors, and prognosis of patients hospitalized in critical care units is limited. The disease needs to be better recognized in the area due to the lack of scientific evidence on the risk factors, aetiology, incidence rate, and result of AKI. As a result, it diminishes government visibility and hampers efforts to provide quality care in the intensive care unit. According to our knowledge, we have found that no study has been conducted in Ethiopia, particularly in the study area. In this retrospective study, we investigated the incidence and predictors of AKI in critically ill patients who were admitted to ICU.

Methods

Study Area and Period

The study was conducted at Wachemo University Nigist Ellen Mohammed Memorial Comprehensive Specialized Hospital, which is located in the Central Ethiopia Region of Ethiopia. It is one of the teaching hospitals in Ethiopia. It is found in Hosanna, which is the capital city of the region, 230 km away from Addis Ababa, and the capital city of Ethiopia. The hospital provides services for more than 5 million people, both outpatients and inpatients, including emergency and intensive care units. The study period was from March 1 to 30, 2023, by reviewing five years of follow-up data on charts of ICU patients who were admitted from September 1, 2018 to August 30, 2022 GC.

Study Design

An institutional-based retrospective follow-up study was conducted among patients who were admitted to the intensive care unit of Wachemo University Nigist Ellen Mohammed Memorial Comprehensive Specialized Hospital.

Source of Population

All patients who were admitted to the Intensive Care Unit of Wachemo University Nigist Ellen Mohammed Memorial Comprehensive Specialized Hospital.

Study population

All adult patients who were selected using a systematic sampling technique in the Intensive Care Unit of Wachemo University Nigist Ellen Mohammed Memorial Comprehensive Specialized Hospital during the study duration were study population.

Eligibility Criteria

All adult patients whose age is greater than or equal to 18 years, admitted to ICU for more than 24 hours from September 1, 2018, to August 30, 2022, at Wachemo University Nigist Ellen Mohammed Memorial Comprehensive Specialized Hospital, and whose chart is complete and available during data collection period were included in the study whereas, patient with a previous history of dialysis, renal transplant, or admission with end-stage renal disease. Patients' records with no baseline records (serum creatinine) were excluded from the study.

Sample size determination and sampling procedure

The sample size was determined by Using Stata statistical software version 14 by applying Cox model assumptions considering Cardiovascular disease as a predictor variable for AKI from a previous study conducted in Ethiopia[27] with an effect size of (hazard ratio) of 1.6. Finally, the required sample size was estimated to be 317 after adding 10% incomplete data. Hence, 317 charts were retrieved. A systematic sampling technique was applied to select charts to be reviewed, including every two charts after determining the first case by lottery methods. The subsequent charts were considered if it appeared that any charts had been missed or had incomplete data.

Operational definition and measurements

Acute Kidney Injury(AKI): Acute Kidney Injury is defined as if one of the following criteria meet; an increase in serum creatinine level by 0.3 mg/dL or more within 48 hours or an Increase in serum creatinine to 1.5 times baseline or more within the last 7 days or Urine output less than 0.5 mL/kg/h for 6 hours using Kidney Disease Improving Global Outcomes (KDIGO) criteria[3]. **The event was** considered when acute kidney injury occurred on follow-up after 24 hours of Intensive Care Unit admission to the end of the study while those patients who discharged without AKI, remained on following during the study period, transferred out, and died without AKI due to other causes during the follow-up period were considered as **censored.** **Survival time** was the time measured in days from the admission of ICU to the development of Acute Kidney Injury or censored. **Baseline period,** which was starting after 24 hours of ICU admission. The **triage score** at admission was determined using the **A**irway, **B**reathing **C**irculation and **D**isability (ABCD) triage score system, which was categorized into three categories: mild (green) if the patient scored between 0 and 4, moderate (yellow) if the patient scored 5-8, and severe (red) if the patient scored greater than 8. For every patient in the critical care unit, we calculated the **cumulative fluid balance** using the percentage (%) from the date of ICU admission until the end of the follow-up (event or censored). Therefore, using the daily cumulative fluid balance (%) subtraction daily input to output in liters, divided by the patient's admission weight and multiplied by 100 for every ICU patient, we classified the patient's fluid balance as positive if the patient recorded a cumulative fluid balance of ≥0% and negative fluid balance if the patient recorded a fluid balance of < 0%[28].

Data collection and procedure

First, the information on the patient charts was reviewed and an appropriate data extraction checklist was prepared in English language. The checklist was adapted from intensive care unit patient monitoring, and

triage sheets, and by reviewing different related articles[7, 12, 20, 25, 29]. The tool comprised socio-demographic details, the date of the patient's admission to the intensive care unit (ICU), the diagnosis, the baseline vital signs, the baseline laboratory results, comorbidities, the medication administered, the vital signs at the end of the follow-up, the laboratory results at the end of the follow-up, complications, the date of the last follow-up (the date of the developing AKI, patient's recovery or death from the ICU). The list of charts was obtained from the database in the electronic recording systems and ICU registration logbooks using the patient's medical record numbers (MRNs). Three data collectors and one supervisor were employed, and data collection was accomplished within a month from March 1 to 30, 2023.

Data quality control

The checklist was pretested on 16(5% of the participants) in randomly selected patient charts to keep data quality before starting the actual data collection period, which were not included in the final analysis. Before data collection began, two days of training about the procedure and tool were provided to data collectors and a supervisor. The data collection procedure was monitored closely by the supervisor and principal investigators daily for completeness and consistency of collected data.

Data processing and analysis

At the end of data collection, the collected data were checked for incompleteness, and then data were entered into Epi-data Manager version 4.6.0.6 and exported to STATA version 14 for cleaning, editing, coding, and analysis. Explanatory data analysis was done to determine missing values, normality tests, and the presence of outliers before data analysis. Then the data were described using relative frequency, percent, mean with standard deviation, and median based on the applicability. A life table was used to estimate the cumulative probability of acute kidney injury at different time intervals. A Kaplan-Meier's survival curve was used to estimate median survival time during the follow-up period and log-rank tests were also used to compare survival curves for the presence of differences in the incidence of AKI among the groups.

The Bi-variable analysis was computed to identify possible associations between AKI and each dependent variable using the Cox proportional hazard model. Variables, significant at $p \leq 25\%$ level in the bi-variable analysis, were collectively included in the multi-variable analysis, to identify independent predictors of AKI.

Using variable inflation factors (VIF) between independent variables, multi-co-linearity was checked, and all outputs fell within the acceptable range (mean VIF= 1.27). The proportional hazard assumptions were also checked using the Global test with a value of $p = 0.1575$, which was insignificant. The Cox-Snell residuals were also used to assess the Cox regression model's fitness to the data. Finally, we could conclude that the final model fits successfully. In multivariable analysis, any statistical was considered significant at $p<0.05$. The association

between the incidence of AKI and independent variables was declared using an adjusted hazard ratio (AHR) with a 95% confidence interval. Finally, data was presented using tables, graphs, and texts.

Ethical consideration

Ethical consideration was obtained from the institutional review board of Wachemo University (protocol number=WCU-IRB 0011/23). After, approval by IRB, official letters of cooperation were written for the hospital administrators. The documentation of informed consent was waived by our institutional review board at Wachemo University. Then, data was collected after getting a permission from hospital administrators on behalf of the patients since the study was conducted retrospectively. Data coding aggregates were used to ensure anonymity and confidentiality.

Result

From the beginning of September 2018 to the end of August 2022, a total of 1058 patients were admitted to the Intensive Care Unit. About 291 charts were excluded using exclusion criteria. Hence, 767 charts were candidates for the review charts, and 317 charts were selected by using a systematic sampling technique to be reviewed, with every two charts included after determining the first case by lottery method. The subsequent charts were considered if it appeared that any charts had been missed or had incomplete data.

Socio-demographic characteristics

In this study, 184(58.0%) of the study participants were male, of which 76(41.3%) of them developed Acute Kidney Injury (AKI) whereas 133(39.1%) female participants developed AKI during their ICU stay. The mean age of the study participants was 54.4 years (SD $\pm$18.19356) with a minimum of 19 years and a maximum of 97 years. Regarding the age categories of study participants, the majority of acute kidney injury, 57(46.0%), were recorded among patients after sixty years of age while a small proportion of AKI, 22(29.7%) occurred among those aged <=40 years (**Figure 1**).

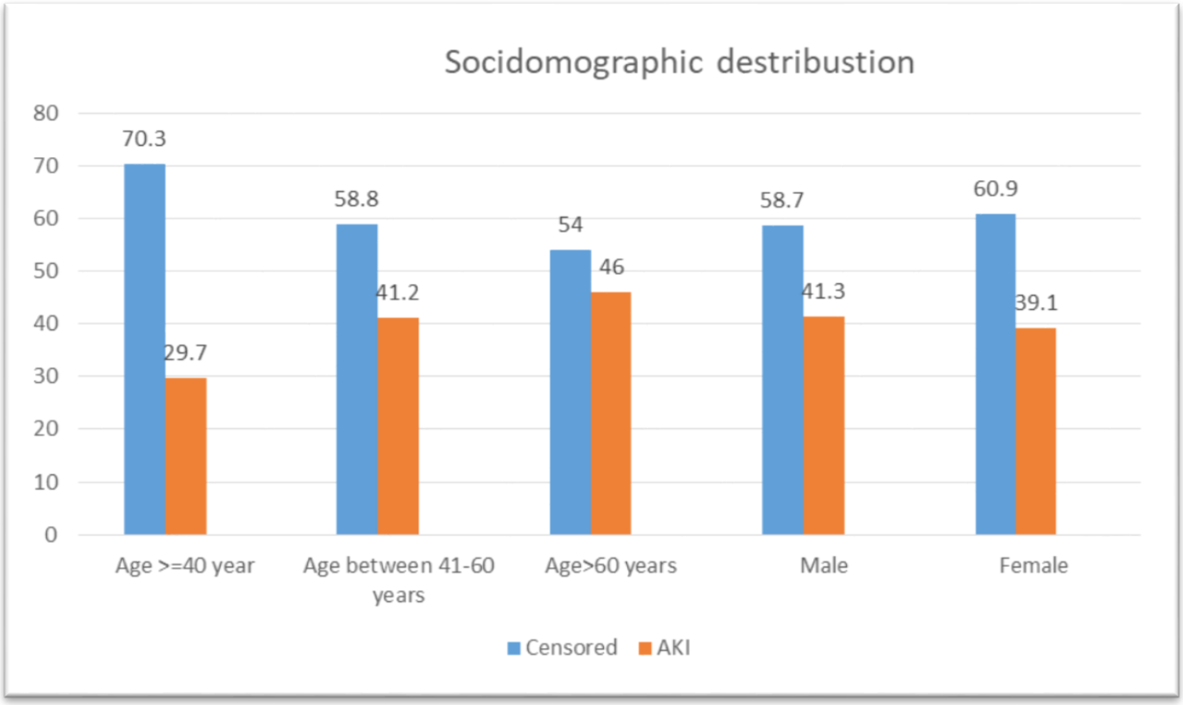

Figure 1: Socio-demographic characteristics of intensive care unit patients admitted to Wachemo University Nigist Ellen Mohammed Memorial Comprehensive Specialized Hospital, Hosanna, Ethiopia, 2023.

Comorbidities and complications of study subjects

Based on the findings, nearly 90% (285/317) of patients were admitted to the Intensive care unit with severe triage scores at admission. The findings showed that 200(63.1%) patients had at least one comorbid condition, of which 46.5% (93/200) had developed AKI. On the contrary, 29.9% (35/117) of the participants who did not have at least one chronic medical illness developed AKI in the ICU. This study's findings revealed that the most prevalent recorded comorbidities were Hypertension 106(33.4%), Diabetics Mellitus 84(26.5%), and Cardiovascular Disease 82(25.9%). The proportion of AKI was much higher among patients having Hypertension (67.9%, n=106) compared to patients who were free of Hypertension (26.5%, n3=211). Furthermore, the study findings revealed that 70.7% of study participants had at least one ICU complication. Hospital Acquired Infections (HAIs) (40.7%, n=317), Acute Kidney Injury (AKI) (40.4%, n=317), Shock (31.2%, n=317), and Delirium (29.3%, n=317) were the most prevalent recorded ICU complications among study subjects. The study findings also showed that the occurrence of AKI among patients who have ICU complications was (46.9%, n=224). Conversely, 24.7 %(23/93) of the study subjects who did not have any ICU complication developed AKI during their ICU stay (**Table 1**).

Table 1: Distribution of Clinical characteristics and comorbidities among patients admitted to the intensive care Unit of Wachemo University Nigist Eleni Mohammed Memorial Comprehensive Specialized Hospital, Hosanna, Ethiopia, 2023 **(n=317)**

Variables	Categories	Outcome Status

		AKI n (%)	Censored n (%)	Total n(%)
Triage Score at Admission	Mild	4(33.3)	8(66.7)	12(3.8)
	Moderate	11(55.0)	9(45.0)	20(6.3)
	Severe	113(39.6)	172(60.4)	285(89.9)
At least one Comorbidities	No	35(29.9)	82(70.1)	117(36.9)
	Yes	93(46.5)	107(53.5)	200(63.1)
Diabetics Mellitus	No	87(37.3)	146(62.7)	233(73.5)
	Yes	41(48.8)	43(51.2)	84(26.5)
Hypertension	No	56(26.5)	155(73.5)	211(66.6)
	Yes	72(67.9)	34(32.1)	106(33.4)
Malignancy	No	120(39.7)	182(60.3)	302(95.3)
	Yes	7(53.7)	8(46.3)	15(4.7)
Stroke	No	116(40.3)	172(59.7)	288(90.85)
	Yes	12(41.4)	17 (58.6)	29(9.15)
HIV/AIDS	No	118(40.8)	171(59.2)	289(91.2)
	Yes	10(35.7)	18(64.3)	28(8.8)
COPD	No	123(41.1)	176(59.9)	299(94.3)
	Yes	5(27.8)	13(72.2)	18(5.7)
Bronchial Asthma	No	123(41.7)	172(58.3)	295(93.1)
	Yes	5(22.7)	17(77.3)	22(6.9)
Cardiovascular disease	No	103(43.8)	132(56.2)	235(74.1)
	Yes	25(30.5)	57(69.5)	82(25.9)
Complication	No	23(24.7)	70 (75.3)	93(29.3)
	Yes	105(46.9)	119(53.1)	224(70.7)
Thromboembolism	No	97(39.75)	147(60.25)	244(77.0)
	Yes	31(42.5)	42(57.5)	73(23.0)
Shock	No	71(32.6)	147(67.4)	218(68.8)
	Yes	57(57.6)	42(42.4	99(31.2)
Acute lung injuries	No	107(40.7)	156(59.3)	263(83.0)
	Yes	21(38.9)	33(61.1)	54(17.0)
Acute Liver Injury	No	111(38.8)	175(61.2)	286(90.2)
	Yes	17(54.8)	14(45.2)	31(9.8)
Hospital Acquired infection	No	41(21.8)	147(78.2)	188(59.3)
	Yes	87(67.4)	42(32.6)	129(40.7)
Electrolyte Imbalance	No	90(37.3)	151(62.7)	241(76.0)
	Yes	38(50.0)	38(50.0)	76(24.0)
Delirium	No	94(42.0)	130(58.0)	224(70.7)
	Yes	34(36.6)	59(63.4)	93(29.3)
Arrhythmia	No	111(40.5)	163(59.5)	274(86.4)
	Yes	17(39.5)	26(60.5)	43(13.6)

Abbreviations: AKI, acute kidney injury; COPD, chronic obstructive pulmonary disease; HIV, human immune Virus; AIDS, acquired immune deficiency syndrome

Management and Laboratory-related Variables

According to the study result, about 54% of the study subjects were intubated, of which (61.45, n=171) developed AKI, whereas the remaining 46 % of the patients were ventilated with Non-invasive ventilation, of which only

15.75 % (23/146) of the participants developed AKI. Regarding the baseline vital signs, 174(55%) of the study subjects were found below 90% peripheral oxygen saturation, of which 52.9% (92/174) of the patients developed AKI. Among patients who had recorded an average respiratory rate equal to or greater than 24 breaths per minute, 42.0 %(105/250) of the patients developed AKI. Regarding the mean arterial blood pressure of the study subjects, 48.8 % (154/3170 of the patients had below 65mmHg, of which 24.7% of the patients developed AKI. Concerning the fluid balance, the majority 252(79.5%) of the study participants had positive fluid balance whereas only 65(20.5%) of the patients had negative fluid balance, of which 60% (39/65) of the study subjects developed AKI. Moreover, the average white blood cell count at baseline was a mean of $12.07x10^3$ c/µl (SD$\pm$5.5) with a minimum of $2.3x10^3$ c/µl and a maximum of $31.1x10^3$ c/µl. Almost two- 74.1 % (235/317) of the study participants had greater than or equal to 150,000 platelet count at baseline, of which 37.9% of those developed AKI as 47.6% of the patients with below 150,000 c/µl platelet count developed AKI(**Table 2**).

Table 2: Distribution of management-related variables among patients admitted to ICU of Wachemo University Nigist Eleni Mohammed Memorial Comprehensive Specialized Hospital, Hosanna, Ethiopia, 2023(**n= 317**).

**Variables**	**Category**	**Outcome** AKI n (%)	Censored n (%)	Total n (%)
Method of oxygen delivery	Non-invasive Ventilation	23(15.75)	123(84.25)	146(46.1)
	Invasive ventilation	105(61.4)	66(38.6)	171(53.9)
Mode of invasive ventilation	VCV/VAC	41(60.3)	27(39.7)	68(39.8)
	PRVC	32(69.60	14(30.4)	46(26.9)
	PCV/PAC	25(64.10)	14(35.9)	39(22.8)
	SIMV	7(38.89)	11(61.11)	18(10.5)
Noninvasive ventilation	CPAP	14(13.3)	91(86.7)	105(71.9)
	NRFM	9(21.9)	32(78.1)	41(28.1)
Average Oxygen Saturation	>=90%	36(25.2)	107(74.8)	143(45.1)
	<90%	92(52.9)	82(47.1)	174(54.9)
Average Respiratory rate	<24b/m	23(34.3)	44(65.7)	67(21.1)
	>=24b/m	105(42.0)	145(58.0)	250(78.9)
MAP baseline	<65mmHg	38(24.7)	116(75.3)	154(48.6)
	>=65mmHg	90(55.2)	73(44.8)	163(51.4)
Fluid Balance	Positive	89(35.5)	163(64.7)	252(79.5)
	Negative	39(60.0)	26(40.0)	65(20.5)
WBC Baseline	<=11,000	57(39.9)	86(60.1)	143(45.1)

	>11,000	71(40.8)	103(59.2)	174(54.9)
	<12 mg mg/dl	25(40.3)	37(59.7)	62(19.6)
Hemoglobin baseline	>=12mg/dl	103(40.4)	152(59.6)	255(80.4)
	<150,000	39(47.6)	43(52.4)	82(25.9)
Platelet baseline	>=150,000	89(37.9)	146(62.1)	235(74.1)
	<1mg/dl	94(35.3)	172(64.7)	266(83.9)
Creatinine level baseline	1-1.2md/dl	34(66.7)	17(33.3)	51(16.1)
	<45mg/dl	99(37.4)	16662.6)	265(83.6)
BUN at the baseline	>=45mg/dl	29(55.8)	23(44.2)	52(16.4)
Serum Sodium at the end of follow-up	<135 mEq/l	45(38.1)	73(61.9)	118(37.2)
	135-148 mEq/l	83(41.7)	116(58.3)	199(62.8)
	<3.5mEq/l	10(35.7)	18(64.3)	28(8.8)
Serum Potassium at the end of follow-up	3.5-5mEq/l	48(27.4)	127(72.6)	175(55.2)
	>5mEq/l	70(61.4)	44(38.6)	114(36.0)
	<=11,000	24(34.3)	46(65.7)	70(22.1)
WBC at the End of follow-up	>11,000	104(42.1)	143(57.9)	247(77.9)

Abbreviations: AKI, acute kidney injury; BUN, blood urea nitrogen; CPAP, continuous positive airway pressure; MAP, mean arterial pressure; Mg, mill gram; NRFM, non-reservoir face mask; PAC, pressure assist control; PCV, pressure control ventilation, PRVC, pressure regulated volume control; SIMV, synchronized intermittent mandatory ventilation; VCV, volume control ventilation; WBC, white blood cells

Description of Drug-related Variables

This study's findings revealed that all 317(100%) of the study subjects took antibiotics. The proportion of AK among patients who received vancomycin 53.9% (104/193) was higher compared to those who did not receive vancomycin 19.5% (24/124) of patients developed AKI. Among the study participants, only 136(42.9%) of the patients received vasopressors, of which 65.4 % (89/136) of the patients developed AKI. In addition, 76.7%, 45.4%, 33.3%, and 28.1% of the patients took thromboprophylaxis, systemic steroids, antihypertensive, and diuretics respectively. Regarding sedation, the majority 60% (189/317) of the study subjects took sedation, of which 56.5% (105/189) of the patients developed AKI (**Table 3**).

Table 3: Distribution of drug Management of patients admitted to the Intensive care unit of Wachemo University Nigist Eleni Mohammed Memorial Comprehensive Specialized Hospital, Hosanna, Ethiopia, 2023(**n=317**).

Variables	Categories	Outcome Status		
		AKI	Censored	Total (%)
	No	68(40.2)	101(59.8)	169(53.3)
Ceftazidime	Yes	60(40.5)	88(59.5)	148(46.7)
	No	24(19.35)	100(80.65)	124(39.1)
Vancomycin	Yes	104(53.9)	89(46.1)	193(60.9)

	No	83(35.9)	148(64.1)	231(72.9)
Meropenem	Yes	45(52.3)	41(47.7)	86(27.1)
	No	123(40.2)	183(59.8)	306(96.5)
Gentamycin	Yes	5(45.45)	6(54.55)	11(3.5)
	No	94(43.9)	120(56.1)	214(67.5)
Ciprofloxacin	Yes	34(33.0)	69(67.0)	103(32.5)
	No	39(26.55)	142(78.45)	181(57.1)
Vasopressors	Yes	89(65.4)	47(34.6)	136(42.9)
	No	66(38.2)	107(61.8)	173(54.6)
Systemic steroid	Yes	62(43.1)	82(56.9)	144(45.4)
	No	28(37.8)	46(62.2)	74(23.3)
Thrombolytic	Yes	100(41.2)	143(58.6)	243(76.7)
	No	72(34.0)	140(66.0)	212(66.9)
Antihypertensive	Yes	56(53.3)	49(46.7)	105(33.3)
	No	109(40.5)	160(59.5)	269(84.9)
Antiviral	Yes	19(39.6)	29(60.4)	48(15.1)
	No	93(40.8)	135(59.2)	228(71.9)
Diuretics	Yes	35(39.3)	54(60.7)	89(28.1)
	No	23(18.0)	105(82.0)	128(40.4)
Sedation	Yes	105(55.6)	84(44.4)	189(59.6)
	No	101(44.3)	127(55.7)	228(71.9)
Ketamine	Yes	27(39.3)	62(69.7)	89(28.1)
	No	115(40.6)	168(59.4)	283(89.3)
Propofol	Yes	13(38.2)	21(61.8)	34(10.7)
	No	103(42.0)	142(58.0)	245(77.3)
Ketofol	Yes	25(34.7)	47(65.3)	72(22.7)
	No	58(28.4)	146(71.6)	204(64.35)
Diazepam	Yes	70(62.0)	43(38.0)	113(35.65)

Abbreviations: AKI, acute kidney injury

Survival function and Incidence rate of Acute Kidney Injury

In this study, the patients were followed for a minimum of 1 day to 48 days, with the median follow-up period being 23 days (95% CI: 18.0, 33.0). The total person-time observation was 4248 person-days. The finding of this study revealed that the overall occurrence of AKI among ICU critically ill patients was 128 (40.4%) (95% CI 35.08 to 45.90), and the rest 189 (59.6) of the participants were censored. Among those considered censored (n=189), 33.4% (106) of the patients died, 22.4% (71) of patients were discharged alive, and the rest 3.8% (12) of the patients transferred to other institutions. The incidence rate of Acute Kidney injury among intensive care unit patients was 30.1 (95% CI: 25.33, 35.8) per 1000 person-days observation. The probability of developing AKI at 1, 24 and 48 days were 0.9874, 0.4728 and 0.1359 consecutively. The cumulative probability of failure at the end of 12, 24, 36, and 48 days were 0.3582, 0.5272, 0.6612 and 0.8641 respectively (**Table 4**).

Table 4: Life table of critically ill ICU patients who were admitted at Wachemo University Nigist Eleni Mohammed Memorial Comprehensive Specialized Hospital, Hosanna, Ethiopia, 2023.

The time interval, days	Beginning total	Fail	Survival probability	Cumulative failure, probability
0-12	317	90	0.4543	0.3582
12 -24	144	24	0.1640	0.5272
24-36	52	10	0.0631	0.6612
36-48	21	4	0.0032	0.8641

Survival status using the Kaplan-Meier Curve

The median time was 23 days. We can note from the graph that at the initial time of diagnosis the probability of developing AKI was lower, but as follow-up time increased the probability of developing AKI also increased (**Figure 2**).

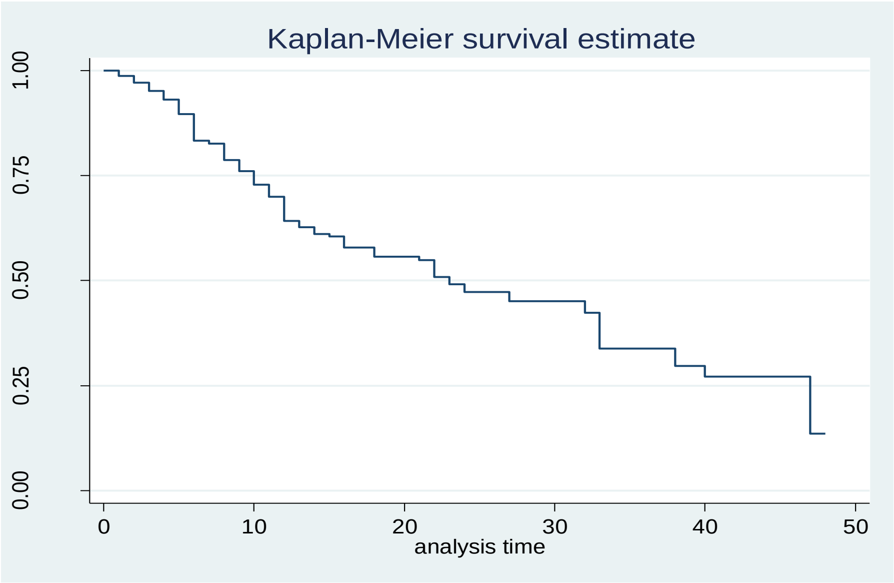

Figure 2: Overall Kaplan-Meier curve for critically ill ICU patients at Wachemo University Nigist Eleni Mohammad Memorial Comprehensive Specialized Hospital, Hosanna, Ethiopia, 2023(N=317). Horizontal axis (X): shows the time of analysis in (days). The vertical axis (Y): indicates survival probability—middle line (downward) survival function.

Survival function and comparison of survivorship function for the fluid balance category

The Kaplan-Meier estimator survival curve gives the estimate of survival function in the fluid balance category to make a comparison. The survivor function line above another means, that the group defined by the upper line curve had better survival than other group line cures within the category. To test the equality of survival curves in Cochrane; the Mantel Henszel log Rank test was performed. The test statistics obtained from the Log Rank

showed there is a statistical difference to test the null hypothesis, which shows there is a difference in the distribution of survival time among categorical variables.

In this study, the negative fluid balance has a higher probability of developing AKI with a median time of 12 days (95% CI; 10-27) as compared with positive fluid balance. At the end of the follow-up, the probability of developing AKI of negative and positive fluid balance was found to be 16.3% and 14.6% respectively. The difference was statistically significant with a p-value of 0.0045(**Figure 3**).

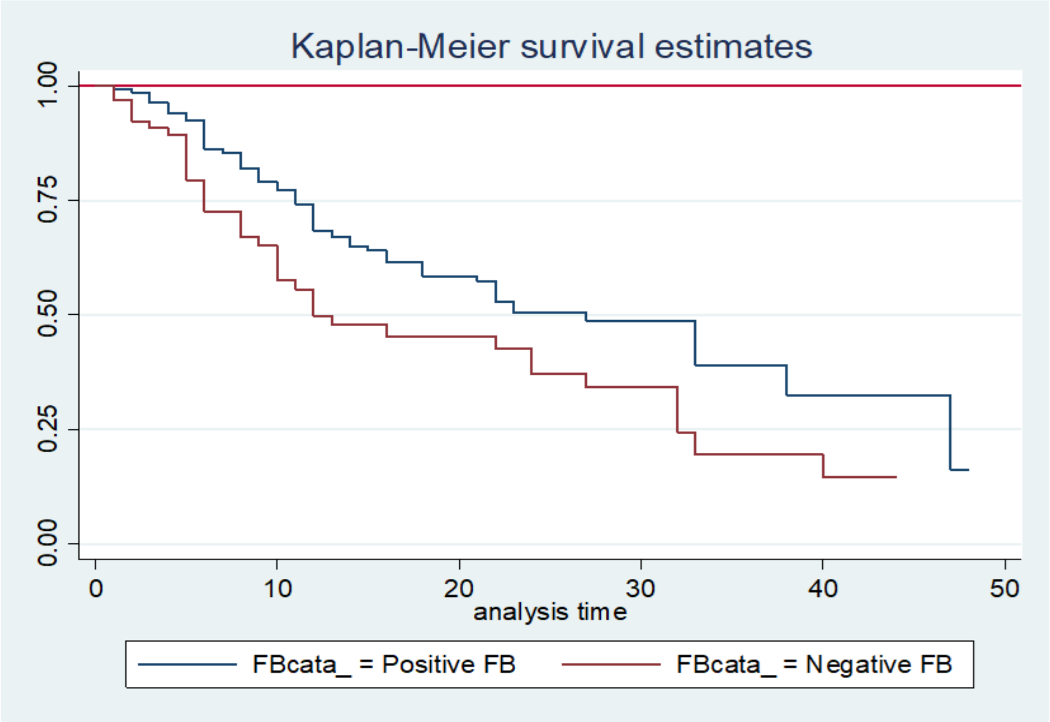

Figure 3. The Kaplan-Meier curve comparison among fluid balance category for ICU patients at Wachemo University Nigist Eleni Mohammad Memorial Comprehensive Specialized Hospital, Hosanna, Ethiopia, 2023(N=317).

Predictors of Acute Kidney Injury among Intensive Care Unit Patients

After determining the nature of the data, the Kaplan- Meier (KM) curve and the life of tables were used for data descriptions. Then, a long-rank statistic test was used to identify the presence of significant differences in mean survival time between groups. Afterwards, a bivariate analysis was conducted using Cox proportional hazard regression to identify variables that had an association with the dependent Variable, and variables to be included in the final model for multivariate analysis were identified. Accordingly, in bivariable analysis, thirteen variables were included, presence of chronic medical, hypertension, method of ventilation, respiratory rate, hospital-acquired infection, Vancomycin, mean arterial blood pressure, sedation, oxygen saturation level, peak inspiratory pressure, positive end-expiratory pressure, vasopressor and fluid balance collectively transferred to multivariate

analysis. However, sedation and oxygen saturation levels were excluded since they violated the proportional hazard assumptions with a value of less than 0.05 during the Schoenfield residual test.

In multivariable analysis, having chronic hypertension, invasive ventilation, negative fluid balance, and vasopressors were found to be independent predictors of Acute Kidney Injury among critically ill patients in ICU at a 95% confidence interval. Patients having hypertension were 1.60 times increased hazard of developing Acute Kidney Injury compared to their counterparts (AHR=1.6; 95%CI: 1.05-2.38). The hazard of AKI was 2.6 times higher among invasive mechanically ventilated patients compared to patients who ventilated with non-invasive ventilation (AHR=2.64; 95% CI: 1.46-4.78). On the other hand, the hazard of developing AKI among patients who have Negative fluid balance in the ICU was twofold higher compared to their counterparts (AHR=2.00; 95% CI: 1.30-3.03). Additionally, patients who took vasopressor while in ICU also revealed statistically a significant association with the occurrence of AKI. The hazard of AKI among patients who took vasopressor was 1.7 times (AHR=1.72; 95%CI: 1.10-2.63) higher than those who did not take vasopressor (**Table 5**).

Table 5: Bivariate and Multivariate analysis of Cox proportional hazard regression among intensive care unit patients admitted in Wachemo University Nigist Eleni Mohammed Memorial Comprehensive Specialized Hospital, Hosanna, Ethiopia, 2023(n=317).

Covariate	Categories	Disease status		CHR 95%CI	AHR 95% CI	Sig.
		AKI	Censored			
	No	35(29.9)	82(70.1)	1	1	
Comorbidities	Yes	93(46.5)	107(53.5)	1.62(1.09-2.39)	0.86(0.561-1.330)	0.524
	No	56(26.5)	155(73.5)	1	1	
Hypertension	Yes	72(67.9)	34(32.1)	3.70 (2.55-5.29)	1.60(1.052-2.384)	0.024*
	NIV	23(15.75)	123(84.25)			
Method of ventilation	IV	105(61.4)	66(38.6)	5.23(3.31- 8.25)	2.64(1.460-4.783)	0.002*
	<24b/m	23(34.3)	44(65.7)			
Respiratory Rate	>=24b/m	105(42.0)	145(58.0)	1.75(1.09-2.79)	1.06(0.6653-1.734)	0.803
Hospital Acquired	No	41(21.8)	147(78.2)			
Infections	Yes	87(67.4)	42(32.6)	3.95(2.71- 7.56)	1.25(0.772-2.016)	0.088
Vancomycin	No	24(19.35)	100(80.65)		1	
	Yes	104(53.9)	89(46.1)	3.94(2.49- 6.21)	1.70(0.983-2.910	0.058
	>=65mmHg	380(24.7)	116(75.3)			
Average MAP	<65mmHg	90(55.2)	73(44.8)	2.51(1.711- 3.69)	1.47(0.981-2.203)	0.058
Average PIP	<35 cmH2O	67(59.4)	132(66.3)			
	>=35 cmH2O	57(59.6)	39(40.6)	1.93(1.35- 2.77)	1.14(0.737-1.761	0.595
Average PEEP	<=10cmH2O	51(26.0)	145(74.0)			
	>10cmH2O	73(75.3)	24(24.7)	3.41(2.37- 4.90	1.54(0.962-2.470)	0.079

	Positive	89(35.5)	163(64.7)			
Fluid Balance	Negative	39(60.0)	26(40.0)	1.72(1.18- 2.51)	2.00(1.301-3.031)	0.004*
Vasopressors	No	39(26.55)	142(78.45)	1		
	Yes	89(65.4)	47(34.6)	3.92(2.68-5.74)	1.72(1.101-2.631)	0.017*

Abbreviation: AKI, acute kidney injury; AHR, adjusted hazard ratio; CHR, crude hazard ratio; IV, invasive ventilation; NIV, non-invasive ventilation; MAP, mean arterial pressure; PEEP, positive end expiratory pressure; PIP, peak inspiratory pressure;

Discussion

This retrospective study aimed to assess the incidence and predictors of Acute Kidney Injury (AKI) among ICU patients. At the end of the follow-up, about 128(40.4%) of the patients developed AKI. The incidence rate of AKI was 30.1 per 1000 person-days of observation. The median time survival time was found to be 23 days (95% CI: 18.0, 33.0). Having chronic hypertension, negative fluid balance, invasive mechanically ventilated, and having taken vasopressor during follow-up were found to be independent predictors of developing AKI. The overall incidence of AKI among ICU patients in this study was 40.4%, which is lower than the findings in China, at 51.0%[16], Tanzania at 55.3%[13], and South Africa at 58.5%[12]. This discrepancy might be due to the difference in sampling method, study areas, socioeconomic status, sample size, and duration of follow-up. However, the finding of this study was higher than the findings of studies in Sudan, 31.6%[14], Egypt 37.4%[15], Cameron, 22.3%[30], and Australia, 5.5%[31]. The variation might be due to the difference in quality care given for ICU, follow-up period, sample size socio-demographic characteristics, and contextual differences among the countries.

Patients having chronic hypertension were found to be at higher hazard of developing AKI by 1.6 times than non-hypertensive patients while keeping other covariates (AHR=1.6; 95% CI: 1.05-2.38). This study was compatible with other studies in Sudan[14], Zimbabwe[32], USA[33], Norway[34], and Ethiopia[35]. The possible justification could be that hypertension in ICU patients under stress results in total peripheral blood vessel resistance, an increase in the amount of extracellular fluid, necrosis of tubular tissue in the nephron, and impaired kidney function, all of which increase creatinine levels, which is the sign of AKI.

The other factor that was independently associated with the development of AKI among ICU patients was the use of vasopressor during the follow-up period. The patients who took vasopressors during follow–up had a 1.7 times higher hazard of developing AKI than those who did not take vasopressors (AHR=1.72; 95%CI: 1.10-2.63). This finding is similar to a study conducted in Tanzania[13], and South Africa[12]. The possible justification could be that the use of vasopressors in critically ill patients may potentially lead to decreased renal blood flow and renal oxygen delivery. This severely impaired renal oxygen due to renal vasoconstriction may cause renal ischemia, resulting in Acute Kidney Injury. However, the data on the effect of vasopressor interventions on renal perfusion

in critically ill patients are not clear. It needs further investigation of the relationship between vasopressors and renal oxygenation.

Moreover, the negative fluid balance was reported as an independent predictor of AKI among critically ill ICU patients. The hazard of developing AKI among patients who have negative fluid balance in the ICU was two times higher compared to their counterparts (AHR=2.00; 95% CI: 1.30-3.03). This finding was contradicted by previous studies conducted in different settings [36, 37]. This discrepancy might be due to the difference in the study population, quality care given in the ICU, follow-up period, sample size, and number of settings. However, this finding is supported by previously reported studies [38, 39] that negative fluid balance harms organ function and reduces long-term survival of mortality in ICU. However, future controlled studies to better investigate the relationship between fluid balance and patient outcomes need to take into account the timing of fluid administration, volume, and types of fluid in critically ill patients.

In this study's findings, the hazard of AKI was about 2.6 times higher among invasive mechanically ventilated patients compared to patients who ventilated with non-invasive ventilation (AHR=2.64; 95% CI: 1.46-4.78). This finding is in similar with studies in Brazil [40, 41]. This might be due to the mechanism of invasive mechanical ventilation attributable to Acute Kidney Injury, including hemodynamic instability and selective renal vasoconstriction by mechanical ventilation-induced sympathetic stimulation. Additionally, invasive mechanical ventilation is attributed to the clinical consequences that lead to altered renal hemodynamics. This leads to hypo perfusion to the kidney tissue due to low cardiac output that impaired venous return and leads to ischemic injury that is termed acute renal tubular necrosis. Finally, this leads to a decline in glomeruli filtration rate and elevation of serum creatinine causing the sign of Acute Kidney Injury.

Limitations of the study

To the best of our knowledge, this study is the first study in Ethiopia to determine the burden of AKI in critically ill ICU patients. This study had its limitations in that we conducted this study in a single center, had a small sample size and also the current study did not evaluate the effect of body mass index, serum albumin, and arterial blood gas analysis since we use secondary data that were not recorded in the charts.

Conclusion and recommendation

The incidence of AKI was high in the study area and was a major public health concern in the ICU. Patients with hypertension, invasive mechanical ventilation, negative fluid balance, and vasopressors were found to be independent predictors of developing AKI in the intensive care unit. It would be better if clinicians in the ICU provided targeted interventions through close monitoring and evaluation of those patients on invasive ventilation, chronic hypertension, negative daily fluid balance, and vasopressors. To the best of our knowledge, this study was the first study in ICU, particularly in Ethiopia. Hence, we recommend that further study be needed to state a

clear direction on the controversial effect of both negative and positive fluid balance on kidney function. Further, clear data will be needed on ventilation parameters that might be factors for AKI.

Implication of this Study

Because it helps with the early identification of AKI risk factors, this is crucial for patients in acute and life-threatening critical care units when making decisions about patient care. According to this study, AKI is a medical problem that is getting worse in the ICU. Early risk factor management has implications for health policy and the provision of high-quality ICU care, and it may transform practice and enhance patient care generally.

Acknowledgements

We would like to acknowledge data collectors, supervisors, staff, and administrators were appreciated for providing the necessary preliminary information. The authors would also like to thank the Wachemo University for giving us this chance.

Funding: This research received funding from Wachemo University since the project was the winner of the 2022 academic year grant.

Authors' contributions: Initially, TMA comes with the study topic. TMA, MSM, AZH, ZDB, and GOB were involved in the study design and tool preparation. TMA wrote the research proposal. YDW, MSM, & and SAJ edited and revised the proposal. Then, TMA, SAS, AZH, GOB, EEE, BB, and YDW participated in the data collection and entry. TMA, AZH, ZDB & MSM conducted the statistical analysis, and TMA drafted the manuscript. MSM, GOB, SAS & EEE edited the manuscript and formatted it for publication. Afterwards, all the authors read, critically revised, approved the manuscript, and agreed to be accountable for all aspects of this research.

Data sharing statement: Extra data that support the findings of this study are accessible from the corresponding author upon reasonable request and can be shared upon legal request via tayemezgebu26@gmail.com

Ethics approval and consent to participate

This study was approved by an institution of review board (IRB) of Wachemo University**.** The study was performed with the declaration of Helsinki. The documentation of informed consent was waived by our institutional review board of Wachemo University.

Consent for publication: Not applicable

Disclosure: The authors declared no conflict of interest and the used references are acknowledged.

Reference

1. Makris, K. and L. Spanou, *Acute kidney injury: definition, pathophysiology and clinical phenotypes.* The clinical biochemist reviews, 2016. **37**(2): p. 85.

2. Kellum, J.A., et al., *Kidney disease: improving global outcomes (KDIGO) acute kidney injury work group. KDIGO clinical practice guideline for acute kidney injury*. Kidney international supplements, 2012. **2**(1): p. 1-138.
3. Group, K., *KDIGO clinical practice guideline for acute kidney injury*. Kidney Int. Suppl., 2012. **2**: p. 1.
4. Hoste, E.A., et al., *Global epidemiology and outcomes of acute kidney injury*. Nature Reviews Nephrology, 2018. **14**(10): p. 607-625.
5. Olowu, W.A., et al., *Outcomes of acute kidney injury in children and adults in sub-Saharan Africa: a systematic review*. The Lancet Global Health, 2016. **4**(4): p. e242-e250.
6. Ahmed, A.M.S. and N.H.M. Eltahir, *Incidence and Risk Factors of Acute Kidney Injury in ICU Patients of Omdurman Teaching Hospital*. Open Journal of Nephrology, 2021. **11**(1): p. 43-57.
7. Khuweldi, M., D. Skinner, and K. De Vasconcellos, *The incidence and outcomes of patients with acute kidney injury in a multidisciplinary intensive care unit in Durban, South Africa*. Southern African Journal of Critical Care, 2020. **36**(2): p. 80-84.
8. Samimagham, H.R., et al., *Acute kidney injury in intensive care unit: incidence, risk factors and mortality rate*. Saudi journal of kidney diseases and transplantation, 2011. **22**(3): p. 464-470.
9. Abebe, A., et al., *Clinical outcome and Predictors of Acute Kidney Injury in Adult Patients Admitted to the Medical Ward of Jimma Medical Center, South West Ethiopia: A prospective Observational Study*. 2021.
10. Banda, J., et al., *Predictors of acute kidney injury and mortality in intensive care unit at a teaching tertiary hospital_ID*. Indian Journal of Critical Care Medicine: Peer-reviewed, Official Publication of Indian Society of Critical Care Medicine, 2020. **24**(2): p. 116.
11. Lewington, A.J., J. Cerdá, and R.L. Mehta, *Raising awareness of acute kidney injury: a global perspective of a silent killer*. Kidney International, 2013. **84**(3): p. 457-467.
12. Aylward, R.E., et al., *Risk factors and outcomes of acute kidney injury in South African critically ill adults: A prospective cohort study*. BMC nephrology, 2019. **20**: p. 1-11.
13. Minja, N.W., et al., *Acute kidney injury and associated factors in intensive care units at a tertiary hospital in northern Tanzania*. Canadian journal of kidney health and disease, 2021. **8**: p. 20543581211027971.
14. Magboul, S.M., B. Osman, and A.A. Elnour, *The incidence, risk factors, and outcomes of acute kidney injury in the intensive care unit in Sudan*. International Journal of Clinical Pharmacy, 2020. **42**: p. 1447-1455.
15. Abd ElHafeez, S., et al., *Risk, predictors, and outcomes of acute kidney injury in patients admitted to intensive care units in Egypt*. Scientific Reports, 2017. **7**(1): p. 17163.
16. Jiang, L., et al., *Epidemiology of acute kidney injury in intensive care units in Beijing: the multi-center BAKIT study*. BMC nephrology, 2019. **20**: p. 1-10.

17. Folkestad, T., et al., *Acute kidney injury in burn patients admitted to the intensive care unit: a systematic review and meta-analysis.* Critical care, 2020. **24**: p. 1-11.
18. de Abreu, K.L.S., et al., *Acute kidney injury after trauma: Prevalence, clinical characteristics and RIFLE classification.* Indian Journal of Critical Care Medicine: peer-reviewed, official publication of Indian Society of Critical Care Medicine, 2010. **14**(3): p. 121.
19. Oweis, A.O., et al., *Incidence, risk factors, and outcome of acute kidney injury in the intensive care unit: a single-centre study from Jordan.* Critical care research and practice, 2020. **2020**.
20. Palevsky, P.M., et al., *KDOQI US commentary on the 2012 KDIGO clinical practice guideline for acute kidney injury.* American Journal of Kidney Diseases, 2013. **61**(5): p. 649-672.
21. Abd ElHafeez, S., et al., *Risk, predictors, and outcomes of acute kidney injury in patients admitted to intensive care units in Egypt.* Scientific Reports, 2017. **7**(1): p. 1-8.
22. Hsiao, C.-Y., et al., *Risk factors for development of acute kidney injury in patients with urinary tract infection.* PloS One, 2015. **10**(7): p. e0133835.
23. Dlamini, T.A., et al., *A prospective study of the demographics, management and outcome of patients with acute kidney injury in Cape Town, South Africa.* PloS one, 2017. **12**(6): p. e0177460.
24. Naicker, S., O. Aboud, and M.B. Gharbi. *Epidemiology of acute kidney injury in Africa*. in *Seminars in nephrology*. 2008. Elsevier.
25. Fenna, K., R.T. Erasmus, and A.E. Zemlin, *Hospital-acquired acute kidney injury prevalence in adults at a South African tertiary hospital.* African Health Sciences, 2019. **19**(2): p. 2189-2197.
26. Kahindo, C.K., et al., *Prevalence and factors associated with acute kidney injury in sub-Saharan African adults: a review of the current literature.* International journal of nephrology, 2022. **2022**.
27. Tassew, W.C., N. Birhan, and Y. Zewdu, *Incidence and predictors of acute kidney injury among newly diagnosed type 2 diabetes patients at chronic follow-up clinic of the University of Gondar comprehensive specialized hospital: a retrospective follow-up study.* Research and Reports in Urology, 2021. **13**: p. 613.
28. Selewski, D.T., et al., *Weight-based determination of fluid overload status and mortality in pediatric intensive care unit patients requiring continuous renal replacement therapy.* Intensive care medicine, 2011. **37**: p. 1166-1173.
29. Mezgebu, T.A., et al., *Risk factors of early mortality among COVID-19 deceased patients in Addis Ababa COVID-19 care centres, Ethiopia.* Plos one, 2022. **17**(9): p. e0275131.
30. Halle, M.P.E., et al., *Incidence, characteristics and prognosis of acute kidney injury in Cameroon: a prospective study at the Douala General Hospital.* Renal Failure, 2018. **40**(1): p. 30-37.
31. Bagshaw, S.M., et al., *Changes in the incidence and outcome for early acute kidney injury in a cohort of Australian intensive care units.* Critical care, 2007. **11**: p. 1-9.
32. Gilbert, A., et al., *Risk factors for development of acute kidney injury in hospitalised adults in Zimbabwe.* Plos one, 2020. **15**(10): p. e0241229.

33. Drawz, P.E., R.T. Miller, and A.R. Sehgal, *Predicting hospital-acquired acute kidney injury—a case-controlled study.* Renal failure, 2008. **30**(9): p. 848-855.

34. Søvik, S., et al., *Acute kidney injury in trauma patients admitted to the ICU: a systematic review and meta-analysis.* Intensive care medicine, 2019. **45**: p. 407-419.

35. Chekol Tassew, W., N. Birhan, and Y. Zewdu, *Incidence and predictors of acute kidney injury among newly diagnosed type 2 diabetes patients at chronic follow-up clinic of the university of Gondar comprehensive specialized hospital: a retrospective follow-up study.* Research and Reports in Urology, 2021: p. 613-622.

36. Weinberg, L., et al., *Associations of fluid amount, type, and balance and acute kidney injury in patients undergoing major surgery.* Anaesthesia and intensive care, 2018. **46**(1): p. 79-87.

37. Hatton, G.E., et al., *Positive fluid balance and association with post-traumatic acute kidney injury.* Journal of the American College of Surgeons, 2020. **230**(2): p. 190-199. e1.

38. Mikkelsen, M.E., et al., *The adult respiratory distress syndrome cognitive outcomes study: long-term neuropsychological function in survivors of acute lung injury.* American journal of respiratory and critical care medicine, 2012. **185**(12): p. 1307-1315.

39. Shen, Y., X. Huang, and W. Zhang, *Association between fluid intake and mortality in critically ill patients with negative fluid balance: a retrospective cohort study.* Critical Care, 2017. **21**(1): p. 1-8.

40. Doher MP, Torres de Carvalho FR, Scherer PF, Matsui TN, Ammirati AL, Caldin da Silva B, Barbeiro BG, Carneiro FD, Corrêa TD, Ferraz LJ, Dos Santos BF. Acute kidney injury and renal replacement therapy in critically ill COVID-19 patients: risk factors and outcomes: a single-center experience in Brazil. Blood purification. 2021 Dec 18; 50 (4-5):520-30

41 Santos LL, Magro MC. Mechanical ventilation and acute kidney injury in patients in the intensive care unit. Acta Paulista de Enfermagem. 2015 Mar; 28: 146-51..

Click here to access/download
Supporting Information
Ethical Approval.jpg
