# Supplementary material for: Incidence and predictors of acute kidney injury among adults admitted to the medical intensive care unit of a Comprehensive Specialized Hospital in Central Ethiopia
Source: PLoS One. 2024 Jun 26;19(6):e0304006. doi: 10.1371/journal.pone.0304006 (PMC11207181; doi:10.1371/journal.pone.0304006)
Supplement: Attachment — Submitted filename: Comment to PLOS AKI.docx [file pone.0304006.s003.docx]

Thank you for the opportunity to read and evaluate the manuscript entitled “**Incidence and Predictors of Acute Kidney Injury among Patients in Intensive Care Unit at**

**a Comprehensive Specialized Hospital in Central Ethiopia: PONE-D-4-02557**”.The article covers an interesting topic and suit for publication. However, it needs some revision before being accepted for publication.

**General comments:**

1. The authors should give line numbers to make the review process easy
2. The manuscript has many editorial problems and needs language editing

**Title:**

3. The title needs a slight modification to indicate that your study populations are adults admitted to the medical ICU.

**Abstract:**

4. Avoid/minimize abbreviations: especially don’t start sentences with abbreviations.
5. Mention how many patients developed AKI from the total sample

**Introduction:**

6. The problem is not well stated. I suggest the authors describe how relevant is AKI as a cause of morbidity and mortality in Ethiopia.

**Methods**

7. The authors describe that they have reviewed five years of follow-up data on charts of ICU patients who were admitted from September 1, 2018, to August 30, 2022, GC. But it will be 4 years from September 1, 2018, to August 30, 2022.
8. The authors should describe how many beds the medical ICU has, the average annual number of patients admitted to the medical ICU, and the staff composition under the study area section.
9. The source and study population should be corrected. The findings of this study will not be generalized for patients admitted to the surgical ICU and should indicate that the source population for this study will be “*all patients admitted to the Medical ICU*…… What the authors describe as a study population is the sample included in the study. The study population will be “*All patients who were admitted to the medical Intensive Care Unit of Wachemo University Nigist Ellen Mohammed Memorial Comprehensive Specialized Hospital during the study period.”*
10. Why do the authors use “admission to the ICU for more than 24 hours” as eligibility criteria? The authors also mention the “**Baseline period,** which was starting after 24 hours of ICU admission.” Don’t you consider clinical characteristics at the time of admission?
11. Do the authors exclude patients for incompleteness of any study variable?
12. The authors should mention all the parameters used to calculate the sample size and should cite the correct reference where the hazard ratios and probability of event are taken.
13. Why do the authors prefer the systematic sampling technique over the simple random sampling technique?
14. Do the clinicians in the study setting use the given operational definition for AKI? A patient with a serum creatinine level of 0.6mg/dl may come with 0.9 mg/dl within 48 hours of measurement. So, are we going to classify this patient as having AKI? Couldn’t there be any other explanation?

**Result:**

15. Sociodemographic factors are overlooked. The authors consider only age and sex. Is there any justification for not considering other sociodemographic factors in the study?
16. The authors have a variable “complication” under the comorbidities and complications section. What kind of complication is it? It should be operationalized.
17. I suggest the authors use another table format that doesn’t have d/t color for d/t categories of a variable. Especially, table 2 is not clear.
18. The authors should differentiate between median survival time and median follow-up time. The report is 23 days for both. But I can see from the life table that the survival probability was about 45% around the 12^th^ day. Please re-check the median survival time.
19. I suggest the authors present the table of log-rank test for all important predictor variables rather than showing the comparison of survivorship function for only the fluid balance.
20. The variable comorbidities entered in the multivariable regression should be operationalized.
21. Vancomycin is usually given for hospital-acquired infection but both hospital-acquired infection and vancomycin were considered as separate variables. Do the authors check the correlation b/n these two variables?
22. In Table 5, the sum of the rows is much above the sample size for variable “Average MAP” and below the sample size for variables “Average PIP” and “Average PEEP”. Check the consistency of the sum of rows and columns anyway.

**Discussion:**

23. The justification given for the differences in the incidence of AKI with other findings should be scientific. Considering sample size, follow-up period, and sampling method as an explanation for the difference doesn’t make sense.
24. The justification for the significant factors seems personal suggestions. The authors should cite scientific evidence for the possible explanations.
25. The authors describe that “this study is the first study in Ethiopia to determine the burden of AKI in critically ill ICU patients.” However, there are some studies in d/t parts of Ethiopia and this is a sign of not acknowledging others effort. Check these papers
